# The complete chloroplast genome of *Aster hypoleucus* (Asteraceae: Astereae): an endemic species from China

**DOI:** 10.1080/23802359.2019.1644219

**Published:** 2019-07-19

**Authors:** Qian Wang, Xiu-Hua Wang, Xiao-Cheng Yang, Long-Fei Fu, Qiu-Ju Gong, Zhi-Xi Fu

**Affiliations:** a Key Laboratory of Eco-Environments in Three Gorges Reservoir Region, School of Life Sciences, Southwest University, Chongqing, PR China;; b State Key Laboratory of Systematic and Evolutionary Botany, Institute of Botany, the Chinese Academy of Sciences, Beijing, PR China;; c University of Chinese Academy of Science, Beijing, PR China;; d College of Environment and Ecology, Chengdu University of Technology, Chengdu, PR China;; e Laboratory of Systematic Evolution and Biogeography of Woody Plants, College of Nature Conservation, Beijing Forestry University, Beijing, PR China;; f College of Life Sciences, Sichuan Normal University, Chengdu, PR China

**Keywords:** *Aster hypoleucus*, chloroplast genome, phylogenomics analysis

## Abstract

This study was the first report about complete chloroplast genome of *Aster hypoleucus* (Asteraceae, Astereae), an endemic species in Xizang (China). The circular whole cp genome of *A. hypoleucus* was 152,300 bp in length, contained a large single-copy (LSC) region of 84,031 bp and a small single-copy (SSC) region of 18,269 bp. These two regions were separated by a pair of inverted repeat regions (IRa and IRb), each of them 25,000 bp in length. A total of 134 functional genes were encoded, consisted of 89 protein-coding genes, 37 tRNA genes, and eight rRNA genes. The overall GC content of the chloroplast genome sequence was 37.3%, and the GC contents of the LSC, SSC, and IR regions were 35.2%, 31.2%, and 43.0%, respectively.

The genus *Aster* is one of the most diverse genera in the tribe Astereae, family Asteraceae, including about 152 species (Nesom 1994). The species of *Aster hypoleucus* Hand.-Mazz. is the shrubby endemic species of the genus *Aster*, and it is narrowly distributed in Xizang Autonomous Region, China. It has ecological and medicinal value in western China. So far, the complete chloroplast genome of *A. hypoleucus* has not yet been published.

Fresh leaves of *A. hypoleucus* were collected from Lang county, Xizang Autonomous Region, China and deposited in Herbarium, Sichuan Normal University, SCNU (specimen no.: Z.X. Fu 1475). High quality total genomic DNA was extracted from ca. 6 cm^2^ sections of the silica-dried leaf using improved Tiangen Plant Genomic DNA Kits, add the 4 μl RNAseA and 20 μl Proteinase K after incubated (65 °C). Total DNA was directly constructed short-insert of 150 bp in length libraries and sequenced on the Illumina Genome Analyzer (Hiseq 2000) based the manufacturer’s protocol (Illumina, San Diego, CA, USA) by ORI-GENE, Beijing. Generally, more than 6 Gb of data was obtained for complete cp genome of *A. hypoleucus*. *De novo* assembled in CLC Genomic Workbench v11 (CLC Bio, Aarhus, Denmark) and consensus sequence in Geneious R11.1.5 (Biomatters Ltd., Auckland, New Zealand) with referenced chloroplast genome sequence of *Conyza bonariensis* (Accession: KX792499) and *Lagenophora cuchumatanica* (Accession: Accession: KX063879). The chloroplast genome was annotated using a web-based annotation program GeSeq (https://chlorobox.mpimp-golm.mpg.de/geseq.html) and editing by manual and imagining with OGDraw v1.2 (Lohse et al. 2013).

The complete chloroplast genome of *A. hypoleucus* (GenBank Accession No. MK290824) was 152,300 bp in length and a typical circular structure, which comprising a pair of inverted repeat (IR) of 25,000 bp divided by a large single copy (LSC) region of 84,031 bp and a small single copy (SSC) region of 18,269 bp (Figure 1). The general G + C content was 37.3% in the whole sequence, while corresponding values of 35.2%, 31.2%, and 43.0% in the LSC, SSC, and IR regions. The whole genome contained 134 genes, including 89 protein-coding genes, eight ribosomal RNA genes and 37 tRNA genes, nevertheless, 114 unique genes, 20 genes duplicated in the IRs. In addition, among the annotated chloroplast genomic sequence, 15 genes possessed only single intron, two genes (*ycf3* and *clpP*) possessed two introns.

**Figure 1.**
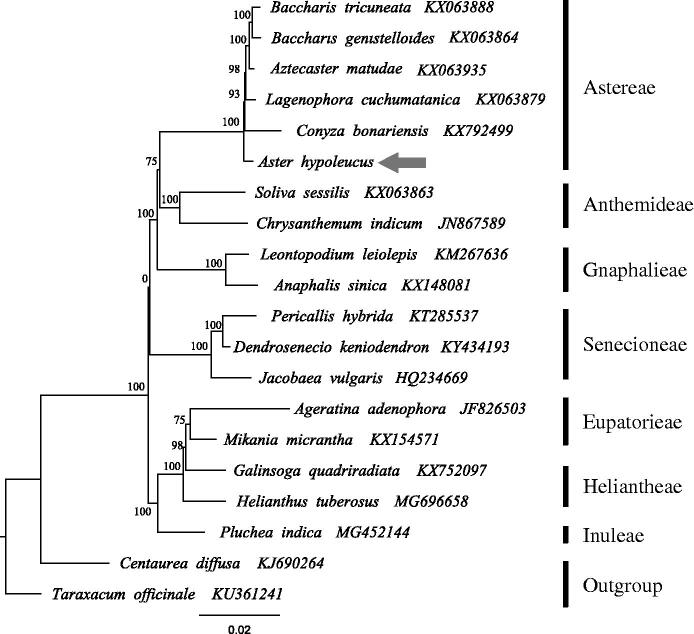
The best Maximum-likelihood (ML) phylogram inferred from 20 chloroplast genomes in Asteraceae (bootstrap value are indicated on the branches). The gene’s accession number is list in the figure.

To construct the phylogenetic tree, all of the cp genome sequences were aligned in MAFFT (Katoh and Standley 2013). A maximum likelihood analysis based on the GTRGAMMA model was performed with RaxML v7.2.8 on the CIPRES (Stamatakis et al. 2008; Miller et al. 2010) using 1000 bootstrap replicates with *Centaurea diffusa* Lam. and *Taraxacum officinale* as outgroup. Phylogenetic analysis shows a well-supported sister-clade relationship *A. hypoleucus* and other members of Astereae (e.g. *Conyza bonariensis*, *Lagenophora cuchumatanica*, and *Baccharis tricuneata*) (Bootstrap support = 100, Figure 1).

The complete plastome sequence of *A. hypoleucus* will provide a useful resource for the conservation genetics of this species as well as for the phylogenetic studies for Asteraceae.
